# Visit to Iraq and Turkey

**Published:** 1956-10

**Authors:** G. Gordon Lennon


### VISIT TO IRAQ AND TURKEY

BY

PROFESSOR G. GORDON LENNON

The Ministry of Health had been asked by the Iraq Government to arrange for a Visiting Professor to go for at least three months to Baghdad. The Ministry requested the Royal College of Obstetricians and Gynaecologists to nominate someone to go and I was invited to do so by the President in May 1955. The University of Bristol, to whom I am grateful, agreed to grant me leave of absence for three months. The British Council in Baghdad arranged for me to go to Turkey on my way home.

I arrived in Baghdad on the 19th January, 1956 at 3.30 a.m. I had left London Airport at 8 o'clock the previous morning, and had come by Amsterdam, Dusseldorf, Vienna and Istanbul (K.L.M.). It was very cold on arrival, cold enough for a hot-water bottle in bed, but this was not forthcoming, so dressing-gown, pyjamas and socks had to do instead! On my way from the airport to the hotel I had envied very much an Arab sleeping in the open by the side of a house accompanied by his donkey and boy. They had a roaring fire nearby. December, January, and the beginning of February can be quite cold, with temperatures even down to freezing. In fact, in Baghdad they say that spring starts on 4th February! Three months later when the weather was beginning to get uncomfortably hot I left. Throughout my stay it had been really pleasant, rather like an English summer with very little rain. The only unpleasant feature was an occasional sandstorm.

### TEACHING THE STUDENTS

The Royal Hospital in Baghdad is the teaching hospital and is situated in the same grounds as the Medical College. There are British professors in all the subjects except obstetrics and gynaecology, and pathology. The medical students are the cream of the Iraqi educational system. The first 100 scholars in the school-leaving certificate who attain 75 per cent. or more are allowed to study medicine if they desire, and they all desire.

During my visit I gave the students lectures twice a week. I took them on ward rounds and gave instruction in the out-patient department. Clinico-pathological discussions were enjoyed most of all. I found the students very intelligent, extremely keen, and possessed of a much better theoretical knowledge than my own students. They were deficient in practical methods largely, I believe, because no one took any trouble to teach them in a practical manner by the bedside. When I say that I instructed the students in the out-patient department it must not be thought that the patients coming to that department were similar to those in our out-patients. There, patients could attend without having been referred by a doctor. Consequently suitable cases for teaching took a little sorting out. As a result work in out-patients tended to be avoided by the senior members of the unit and was left to the most junior members. One can appreciate, then, that the students were most grateful and intensely interested when I took them for teaching in a side room in the out-patient department and discussed methods of examination with them. Only about one in four of the out-patients who were referred to me would consent to be examined but, this difficulty apart, we enjoyed the sessions there.

### PATIENTS

I also operated both on obstetric and gynaecological patients, indeed I covered all aspects of gynaecological operating, and was the first to do Wertheim's operation in Baghdad. The patients walk to the operating table, and nearly all have spinal anaesthesia! The after-care of the operated patient is poor, for example the house surgeons have to do catheterizing if it is necessary. There were many cases of vesico-vaginal fistula, and it was impossible to arrange for suction drainage of the bladder after operation in the management of these cases. All that one could do was to put in a catheter which drained directly into the bed. In spite of this, cases on which I operated were successful: the resilience and powers of recuperation of Iraqi women were truly great!

In the opinion of these people British medical standards and particularly nursing service are supreme. If they have to have a surgical operation many come to England if they can afford to. Consequently I was asked to see many cases in private consultation; but I did few operations outside the Royal Hospital. However, the Iraqis are very keen to improve matters and they have the money to do so.

### BOOM AND PROGRESS

About one hundred million pounds a year are obtained from oil royalties, and the Iraq Government has formed a Development Board to sift requests, to make grants, and to arrange for improvement schemes to be carried out. As a result developments are proceeding in all branches. In fact at the moment Baghdad is a boom city; moreover these improvements are not confined to Baghdad—the rest of Iraq sharing them. Thus while I was there, two bridges were being built over the Tigris. This river divides Baghdad in two. These bridges were being built by German contractors with British consultants. In order to prevent floods the River Tigris has been dammed for the first time by mechanical means by the construction of a dam one hundred miles north of Baghdad. This scheme cost fifteen million pounds and I was honoured to be invited to the opening ceremony and saw the overflow water was being diverted for irrigation of large areas of land north of Baghdad. This dam had also been built by German labour with British consulting engineers.

Naturally, medicine is obtaining its share of funds from the Development Board and the most important project at the moment is the building of a new Royal Hospital in Baghdad at a cost of about five million pounds. Other hospitals are being built elsewhere, particularly for the treatment of tuberculosis, and it is possible that there will be a second Medical School at Mosul in northern Iraq. Baghdad and Mosul are connected by railway and there are the most comfortable air-conditioned sleepers for the night journey from one city to the other. Small private hospitals were mushrooming everywhere, probably because the hospital consultants felt a lack of security in their hospital appointments and because they were not allowed to charge fees in the private wing of the Royal Hospital.

### HISTORICAL BACKGROUND

One would not go to Iraq without visiting many places famous from Biblical times, such as Babylon and Nimrud. At Nimrud I visited the diggings of Professor Mallowan and there saw the most wonderful marble columns. It was interesting to have coffee with the Professor and his wife (Agatha Christie): for one had a feeling that, during the conversation, she was planning the twist in her next murder story or play!

Travel to such ancient places naturally sets one thinking. Here is a people—one might call them the early birds of the dawn of civilization—who have declined, and had been dominated by the Ottoman Empire for four hundred years until as recently as 1917 when the British liberated them, who have been under British mandate until 1932, and who have since then begun to realize their freedom. They are an immensely intelligent and clever people, who only in the last six years have found themselves rich due to oil, and one cannot help but sense the prevailing mood of nationalism. The irrigation schemes alone should make the country in years to come much more fertile and able to support a larger population than the five million at present. They have a past from which we have learned, and they have also a past from which they themselves have learned, particularly of oppression; but they have a great future which they are just beginning to appreciate. They are a happy people, easy to get on with, perhaps too frustrating in their administration—a fault not unknown in this country!

### GOOD BRITISH INFLUENCE AND POOR SALESMANSHIP

It was valuable to have time to see one's own country from a distance. In this boom city there was healthy British influence in engineering and in culture. But in trade we are lagging. In Britain we are constantly being urged to export, but when one finds that British exports are not backed up by adequate servicing facilities for the goods in question one realizes the deficiencies in our salesmanship. We export thirty million pounds worth of drugs every year, yet an agent in Baghdad (an Iraqi) told me that he wanted to sell more English drugs, but the British firms did not appreciate the intensity of competition, consequently most of the drugs used in Baghdad and Iraq are American, French, Swiss and German. In Mosul a complaint was made to me regarding the delivery time of surgical instruments. The Iraqis said that the delay was caused not by the surgical firms but by the British Crown Agents through whom the goods had to go. I broke my fountain-pen nib and when I went to the agency in a large store in Baghdad I was told that they only had new pens, and no spares. I should have thought there was nothing easier to export than fountain-pen spare parts! Nor had any of the five big shops in Baghdad even heard of British "Biro", whereas every second shop sold American pens of every description. So I had to buy an American indelible pen. Apparently the Iraqi are able to afford the dollars for American motor cars, although Iraq is a sterling area. Many of them told me that they would prefer British cars, but the servicing and spares' supply were very poor compared with American or German facilities. The German Volkswagen is very popular although it is not the car for the rough desert roads.

### TURKEY

I visited Turkey on my way home and gave lectures at Ankara and at Istanbul. Both these cities are immensely attractive, Istanbul more so because of its beautiful situation, with the Bosporus, the Golden Horn, and the Sea of Marmora. Indeed, in Istanbul one can stand in Europe and look at Asia, and also stand in Asia and look at Europe. One can sit on the terrace of an hotel on the European side of the Bosporus and view the Selimye Barracks on the Asian side, and ponder its associations with Florence Nightingale. One did not feel so much at home in Turkey as one had in Iraq because the language of the Medical School in Iraq is English, whereas the universal language in Turkey is French. So in Turkey I had to have an interpreter for my lectures, and in an hour's lecture one only delivers half an hour's content. There were other interesting experiences in Istanbul—a Press Conference with its awkward questions on painless child-birth and birth control, and a cocktail party which was held by the representative of the British Council in honour of a visiting radiotherapeutist from England and myself. To this cocktail party the Russian Consul had brought a book on "*L'analgésie de l'enfantement*" for me and a book on cancer treatment for the radiotherapeutist. This was the first time I had met Russians in my travels.

Despite inflation the Turks are planning new hospitals and I saw in Ankara a new hospital about to be opened which had been built by the Government as a hospital for private patients because the doctors in the Health Service had no private beds anywhere in Ankara. At this hospital the Government had built a most interesting Nurses' Training School, complete with gymnasium for the nurses, library, and the other usual facilities. This was co-operation between private enterprise and National

Service which interested me very much. In Ankara is one of the best hospitals for obstetrics and gynaecology I have ever seen. The only unsatisfactory feature of the hospital was a radiotherapy machine made by an English firm. The apparatus had never worked since its installation three years previously! Small wonder that the other hospitals being built in Ankara and Istanbul were being equipped with German material.

### THE FUTURE

It is always difficult to assess what one has accomplished on such a trip. One makes many friends, but I also hope and believe that my talks to the students on maternity services, maternal mortality rate and infant mortality rate, may have fallen on fertile ground, and will help the future of the services in Iraq. I sent a report to the Minister of Health which he had asked me for. In it I stressed Iraq's need for an adequate nursing service and the proper training of midwives. Only about 5 per cent. of confinements in Iraq is institutional, and therefore pre-natal care should be all the more intense. But unfortunately at the moment there is little or no pre-natal care. There is a wonderful opportunity for a few British midwives and other specialists to go to Iraq and undertake the building-up of such services, but those who go must look upon the venture as a long-term project and a career, and must dedicate themselves to it. The World Health Organization is attempting to do this, but I feel the task would be both better done and more appreciated through separate individual appointments. Britain's future now depends upon such cultural exchanges: the opportunity exists, and if we in Britain do not accept such a challenge then others will. President Eisenhower has already indicated that the Americans are ready.

